# Biocontainment of Genetically Engineered Algae

**DOI:** 10.3389/fpls.2022.839446

**Published:** 2022-03-02

**Authors:** Jacob Sebesta, Wei Xiong, Michael T. Guarnieri, Jianping Yu

**Affiliations:** Biosciences Center, National Renewable Energy Laboratory, Golden, CO, United States

**Keywords:** biocontainment, algae, cyanobacteria, synthetic auxotrophy, lethal genes

## Abstract

Algae (including eukaryotic microalgae and cyanobacteria) have been genetically engineered to convert light and carbon dioxide to many industrially and commercially relevant chemicals including biofuels, materials, and nutritional products. At industrial scale, genetically engineered algae may be cultivated outdoors in open ponds or in closed photobioreactors. In either case, industry would need to address a potential risk of the release of the engineered algae into the natural environment, resulting in potential negative impacts to the environment. Genetic biocontainment strategies are therefore under development to reduce the probability that these engineered bacteria can survive outside of the laboratory or industrial setting. These include active strategies that aim to kill the escaped cells by expression of toxic proteins, and passive strategies that use knockouts of native genes to reduce fitness outside of the controlled environment of labs and industrial cultivation systems. Several biocontainment strategies have demonstrated escape frequencies below detection limits. However, they have typically done so in carefully controlled experiments which may fail to capture mechanisms of escape that may arise in the more complex natural environment. The selection of biocontainment strategies that can effectively kill cells outside the lab, while maintaining maximum productivity inside the lab and without the need for relatively expensive chemicals will benefit from further attention.

## Introduction

Genetic modification of algae, including eukaryotic microalgae and cyanobacteria, is expected to facilitate direct conversion of light energy and inorganic carbon to a wide variety of valuable chemicals (Angermayr et al., 2015; Gomaa et al., 2016; Santos-Merino et al., 2019; Arora et al., 2020). As with other genetically modified organisms (GMOs), the environmental risk of large-scale cultivation must be assessed, and appropriate measures must be taken to mitigate those risks. Previously, Henley et al. (2013) reported a risk assessment for genetically engineered microalgae (Henley et al., 2013), finding that risks to human health, the environment, and the economy, were generally low, but not zero. Given that genetically engineered algae may be grown outdoors, possibly in open ponds, they determined that the potential for these cells to escape into the environment is elevated beyond that of typical industrial microbial cultivation. Henley et al. (2013) therefore, recommended the development of biocontainment strategies which reduce growth fitness in the natural environment, that are conditionally lethal to the cells when they are not in the lab or industrial setting, and that have reduced capability to transfer genetic material to other organisms. Since that report, many new genetic biocontainment strategies have been developed for microalgae and other industrially relevant microorganisms which achieve one or more of those aims (reviewed by Lee et al., 2018; Wang and Zhang, 2019; Kim and Lee, 2020; Arnolds et al., 2021; Kallergi et al., 2021). In synthetic auxotrophy, cells are modified to make their growth dependent on an unusual or nonnatural nutrient or an unnaturally high concentration of a nutrient. Examples include dependence on unusual phosphorous sources like phosphite (Motomura et al., 2018) and dependence on high concentrations of carbon dioxide (Clark et al., 2018; Lee et al., 2021). Further efforts have been made to express toxic proteins, such as nucleases, in the cells in a manner dependent on the conditions outside the lab, typically, the loss of some synthetic signal molecule or an unnatural concentration of a signal molecule. Biocontainment strategies have been collected in the Biocontainment Finder on the Standardsinsynbio.eu website.

This review will summarize the rationale for designing genetically encoded biocontainment systems and the efforts made thus far to assess their efficacy in genetically engineered algae. First, we discuss the possible escape routes and fates of escaped algae. The regulatory requirements for outdoor cultivation of genetically engineered algae in a few regions are then summarized. Next, an overview of the different types of genetically encoded biocontainment strategies that may be used in algae is provided. We examine whether lab tests, which frequently demonstrate the achievement of meeting the NIH guideline of a 10^−8^ cell survival rate (USA Department of Health and Human Services: National Institutes of Health, 2019), are truly representative of what may occur if cultures were released into the natural environment. Finally, we discuss the results of some specific examples of genetically encoded biocontainment found in recent publications and finish by suggesting future directions.

### How Might GE Algae Escape? What Are the Consequences of Escaped GE Algae?

Biological invasions may proceed through different stages of escape, including proliferation, spread, and persistence. Invading organisms often die out, but in some cases may “alter fundamental ecological properties such as the dominant species in a community and an ecosystem’s physical features, nutrient cycling, and plant productivity” (Mack et al., 2000). We focus first on dispersal and how algal cultivation is likely to differ from that of heterotrophs. Heterotrophic microbes are generally grown in fermenters, inside buildings, with little exposure to the environment. In this situation, escape is most likely to occur *via* discharge of spent growth media with imperfect prior removal of the microbes. Large accidental spills from fermenters or the harvesting equipment may potentially flow out of buildings or greenhouses. At smaller scales, microbes can also hitch a ride on any equipment or workers in contact with the culture. Algae grown at an industrial scale are likely to grow outside, possibly in open ponds, to take advantage of the free energy source provided by sunlight. This direct exposure to the environment presents challenges in terms of the lack of control over conditions such as temperature and light intensity, as well as the significant problem of biological contamination. Competition from natural algae may reduce yields of the desired product and predators may quickly devour the cultivated species (US DOE EERE BETO, 2021).

To prevent genetically engineered algae (GE algae) grown outdoors from leaving the ponds, regulatory agencies in the United States and Mexico have required secondary containment, such as earthen berms, around the ponds to prevent spills from leaving the facility. Further, netting has been required to prevent birds, small mammals, and insects including aphids from entering the pond and potentially carrying away algae to another location (Szyjka et al., 2017; González-Morales et al., 2020). Regulatory agencies may consider enclosed bioreactors differently from open ponds. However, there is likely a heightened risk of glass or plastic bioreactors breaking if they are located outside, compared with those located indoors.

In contrast, regulatory agencies have focused less on whether GE algae can establish themselves in the natural environment once they have escaped the cultivation system. It is generally expected that GE algae are poorly suited for growth in the environment. Some species which are considered model species have been grown continuously in laboratories and may have evolved or acclimated to the favorable environment of the lab where they typically have media much richer than anything found in the environment, they are not exposed to UV light, and where predators and competitors are carefully excluded by researchers. Cells engineered to produce large quantities of valuable products may be further disadvantaged by the metabolic burdens imposed in generating those products. It is not clear, however, that this common conception has been tested by examining the growth of such organisms in natural conditions. In addition, reduced growth rates do not preclude the persistence of escaped cells in the environment. As others have concluded, the risk of GE algae growing in the environment is not zero (Henley et al., 2013).

In addition to the potential for growth at some low rate, escaped cells may continue to exist in a state of persistence. Some bacteria can form spores to persist in environmental conditions unfavorable for growth. Even bacteria that cannot form spores may enter a state of low growth to persist in nutrient-limited conditions (Gray et al., 2019). The persistence state may allow cells time to mutate any toxic genes used for biocontainment, and thus escape. The assays commonly used to assess escape frequency, such as growth curves and colony counting, may not capture this mechanism since the extremely slow growth of the persistent state may appear the same as cell death.

**Figure 1:**
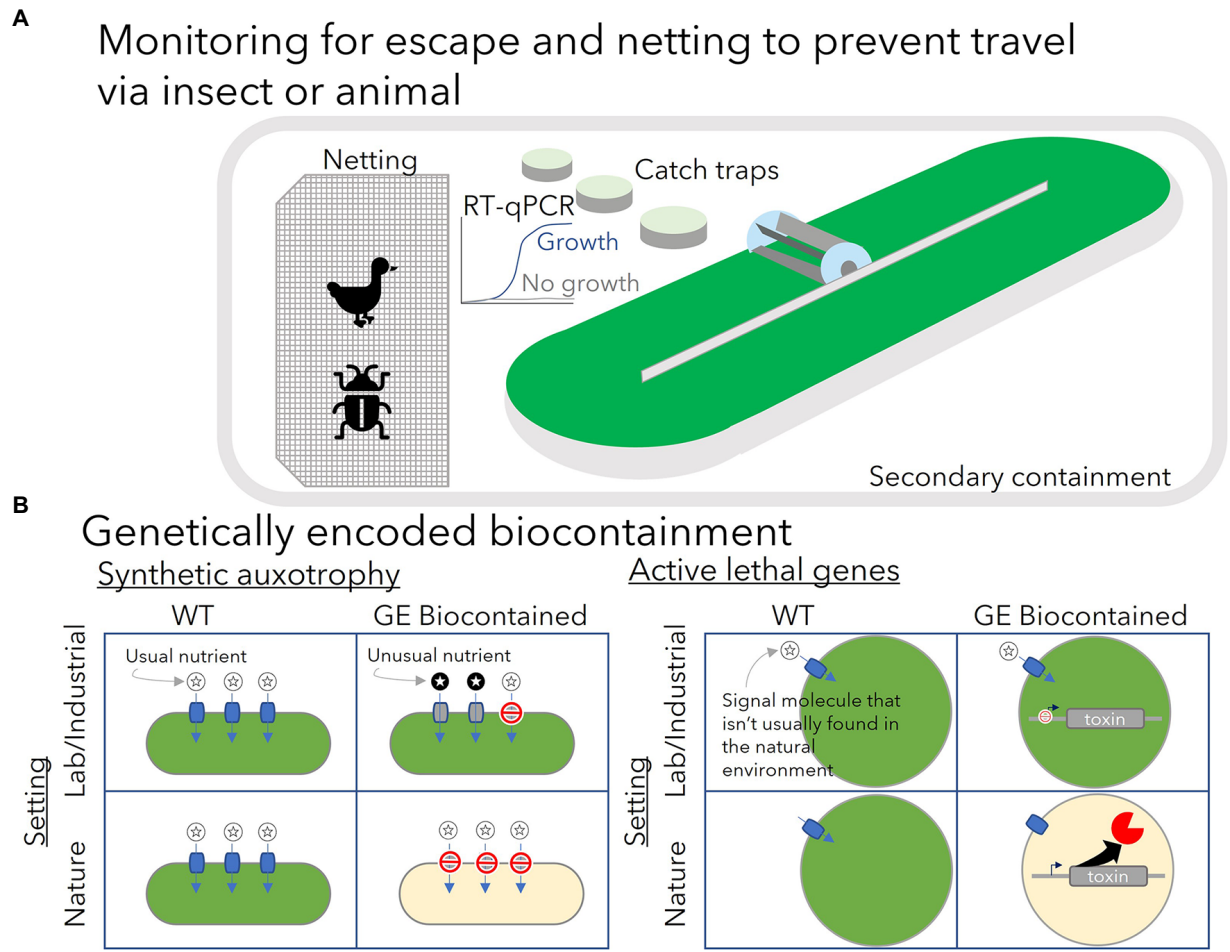
**(A)** Present regulatory requirements for outdoor cultivation of genetically engineered algae have focused on the use of netting and secondary containment such as earthen berms to prevent physical escape and monitoring for escape with nearby catch traps. Catch traps are monitored for growth of the GE algae species grown in the main cultivation system using quantitative PCR. **(B)** Genetically encoded biocontainment is expected to provide another layer of security to prevent growth of GE algae outside the lab or industrial setting. Synthetic auxotrophy creates a growth dependence on an unusual nutrient (dark-circled stars) or an unusually high concentration of a nutrient not likely to be found in nature. Modifications to prevent growth on the usual nutrients (light-circled stars) found in the environment (e.g., knockout of transporters for the usual nutrient—red do not enter symbols) may be necessary to enforce this dependence. Growth can proceed normally in lab or industrial setting when the unusual nutrient is provided in sufficient quantity. In the natural environment, the GE biocontained cells cannot utilize the usual nutrient and cannot grow. Active biocontainment strategies rely on a signal molecule (light-circled star) that is not likely to be found outside the lab/industrial setting to control expression of a toxic protein. Expression of the toxic gene (e.g., a nuclease) is repressed by the signal molecule which can be provided in the lab or industrial setting. Induction by the signal molecule’s absence in the natural environment results in cell death.

If GE microbes escape physical containment, establish themselves, and persist in the natural environment, what harm may be done? A report commissioned by the government of Netherlands summarized possible risks of escape (Enzing et al., 2012). GE microbes that escape compete with native species for nutrients. They may continue producing the valuable products they have been engineered to make, in turn impacting microbial community dynamics. In some cases, those products may have some toxicity to other organisms. The engineered organisms may themselves become food for other organisms which may alter the environment in an unpredictable way. GE algae may contribute to worsening eutrophication, or enriched nutrients in natural water bodies, which can lead to the reduction of dissolved oxygen that may follow an algal bloom. Horizontal gene transfer from GE algae to other organisms could also result in the further spread of antibiotic resistance genes as these genes are often used as selection markers for genetic modification. Ideally, all of these possible ecological disruptions should be avoided. Figure 1 summarizes some of the possible regulatory requirements for outdoor growth of GE algae (A) and the two types of genetically encoded biocontainment strategies that have been demonstrated in lab tests for GE algae (B).

### Recent Field Tests and Regulatory Requirements for Outdoor Growth of GE Algae

The legal status of GE algae (and genetically engineered organisms in general) in the United States has been reviewed in 2014. The authors focus on the challenges for regulating modified microbes with one of the most difficult challenges being in the research and development stage, when the hazards of deployment may not be known until the research is completed (Mandel and Marchant, 2014). Some regulations may apply depending on what genetic modifications were made and how the modified organism will be used. The Federal Insecticide, Fungicide, and Rodenticide Act (FIFRA) may apply to microorganisms intended to be used as pesticides. The FDA regulates microbes that alter the nutrition of food (including if the algae are the food/drug/nutrition supplement) and the Federal Food, Drug, and Cosmetics Act may apply depending on the product application.

In a few cases in the United States and Mexico, companies have discovered what the environmental regulations entail for pilot-scale plants growing GE algae. In the United States and Mexico, environmental agencies have focused first on reducing the chance that the GE algae can leave the cultivation system. For open ponds, this has meant utilizing secondary containment berms around the ponds, netting to avoid contact between the culture and insects and animals, and monitoring of traps arranged outside of the culture vessel or pond to detect escape *via* wind carried water droplets. We next review some details of the permitting process for a pilot plant proposed by the algae biotechnology company, Algenol, in the United States as it has been well documented.

Algenol previously pursued modification of a proprietary cyanobacteria species, called AB1, to produce ethanol (Legere et al., 2017). In 2009, Algenol obtained a determination from the USDA Biotechnology Regulatory Services of the Animal and Plant Health Inspection Service (APHIS) that the strains they utilized were not pathogenic for plants, animals, or humans, and they were therefore not regulated under the Plant Protection Act. A permit was still needed under APHIS for interstate transport of hybrid algae. Research and development activities conducted inside a structure by Algenol were also exempted from permitting under the Toxic Substances Control Act (TSCA). GE algae grown outdoors generally require TSCA Environmental Release Applications (TERA). Since Algenol proposed to cultivate GE algae in closed photobioreactors, the EPA indicated that the facility would be exempted from the TERA application process because they were considered a “contained structure” under the Code of Federal Regulations (CFR) 40 CFR 725.234.^1^ Forty CFR 725.3 simply defines “structure” as “building or vessel which effectively surrounds and encloses the microorganism and includes features designed to restrict the microorganism from leaving.” The Environmental Protection Agency (US) provides specific guidance for TSCA application for algae in the document “Algae Supplement to the Guidance Document: Points to Consider in the Preparation of TSCA Biotechnology Submissions for Microorganisms.”^2^

More recent examples of permitted outdoor cultivation of GE algae at facilities operated by Sapphire Energy in California, United States, and StelaGenomics México in Guanjuato, Mexico are referenced in recent publications. The requirements of the TERA for Sapphire Energy included maintaining secondary containment, netting, and monitoring bucket traps outside the main cultivation pond for growth of the GE algae (Szyjka et al., 2017). Similar requirements were made in Mexico under the Biosafety Law of GMOs (González-Morales et al., 2020). A strain of *Acutodesmus dimorphus*, modified to increase fatty acid synthesis, was grown at a Sapphire Energy pilot plant in California. Traps outside the pond were filled with tap water supplemented with algae growth medium. qPCR and metagenomic analysis were used to monitor the growth in the traps. Very low abundance of the GE strain was found. The wild-type (WT) *Acutodesmus dimorphus* was relatively high in abundance in the traps leading the authors to speculate that the WT may have arrived from the surrounding environment since it is known to have natural abundance in the vicinity. This work also examined the invasion potential of the GE *A. dimorphus* compared with the wild type by inoculating water collected from nearby lakes with each strain. Both strains were found to have minimal impacts on the natural diversity of the lakes. Similar monitoring was done by González-Morales et al. (2020) to comply with regulations for biosafety, in Mexico. Tap water supplemented with BG11 media was placed in traps outside the main cultivation site (a shallow pond with a paddlewheel in a racetrack configuration or raceway pond) between 3 and 28 m distant. PCR and RT-qPCR did not amplify genomic DNA from the engineered *Synechococcus elongatus* PCC 7942 grown in the raceway. In these two cases, transfer of GE algae to the surroundings was minimal.

In Netherlands, another algae biotechnology company, Photanol, has operated a pilot plant and demonstration plant in Amsterdam and Delfzijl, which cultivate genetically modified cyanobacteria. The Dutch government requires permits for contained use and introduction into the environment of GMOs. Market applications of such organisms require a third permit. In the European Union, member nations implement the directives of the European Commission (EC). According to a report commissioned by Netherlands Commission on Genetic Modification, EC directives 2009/41/EC and 2001/18/EC require regulation of contained uses, which generally may apply to GMO algae/cyanobacteria grown indoors (2009/41/EC) and those which may be grown outdoors, including those grown in sealed photobioreactors if they are outdoors (2001/18/EC). For both, an environmental risk assessment is required. However, the facilities can be exempted from risk assessment if the cultivation system has a history of safe use under “good industrial large-scale practice” (GILSP), and the particular GMO is composed of a non-pathogenic host, a “safe” vector, and insert, and the resulting GMO has a lower fitness in the environment than the wild-type host organism (Enzing et al., 2012).

### Overview of Strategies for Genetically Encoded Biocontainment

To address the risks associated with outdoor growth of GE algae, several genetically encoded biocontainment systems have been developed. General strategies have included developing synthetic auxotrophy (Figure 1B) where cell growth is made dependent on some unusual nutrient, and genetic circuits that can sense a change in environmental conditions that indicates the cell has left the controlled conditions of the lab and induce expression of toxic proteins or suppress expression of essential genes (Arnolds et al., 2021).

Synthetic auxotrophy may be conferred by knockout of genes required for nutrient utilization combined with the introduction of genes needed to utilize some unusual nutrient that the cells are unlikely to encounter outside of the lab/industrial setting. One advantage of this approach is that it may be less likely for cells to mutate to regain the ability to utilize more common nutrients. Unlike inducible lethal genes, this does not rely on a signaling pathway in which a mutation in any component may result in failure, and the continued growth of the cells in the environment. One example of this is found in work that has introduced genes needed for phosphite uptake and utilization combined with the knockout of the phosphate transporters (Motomura et al., 2018). A slightly different approach was taken by researchers who knocked out the carbon concentrating mechanism (CCM) genes from a cyanobacterium, resulting in a strain that was dependent on high CO_2_ concentrations for growth (Clark et al., 2018). Both studies were able to demonstrate hypothetical compliance with the NIH’s escape frequency guideline of one in 10^8^ when the cells were grown in zero phosphite and ambient CO_2_ concentrations, respectively. A related strategy for biocontainment may be the utilization of organisms that have already evolved to survive in uncommon environments including extremophile species, such as members of Cyanidiales which can only grow in acidic environments.

Biocontainment systems which are dependent on the absence of the unusual nutrients should carefully consider whether alternative nutrients may be available in some environments. For example, organic carbon sources may be utilized by some algae which would reduce the efficacy of the high inorganic carbon requirement of the system described above. Phosphite-dependent strains may be able to take advantage of naturally occurring phosphite. One lake in eastern China was found to have 1.58 μg/kg phosphite near the surface, which represented about 5.51% of the total phosphorous (Han et al., 2013). Analysis of freshwater from samples in six locations in Florida, United States showed that phosphite and hypophosphite frequently represented more than 25% of the dissolved phosphorous (Pasek et al., 2014). The highest concentration of phosphite measured in that report was approximately 0.1 mM. Bacteria capable of utilizing reduced phosphorus are widespread (Stone and White, 2012), suggesting that phosphite and hypophosphite are common in the environment.

Active biocontainment strategies are distinguished from passive strategies by the utilization of lethal genes, which are induced by a change in the concentration of a signal molecule that the cells are expected to experience if they were to escape from their normal cultivation media into the natural environment (Figure 1B). Inducible promoters are typically required to control expression of lethal genes. However, the options for well-characterized inducible promoters are limited in algae. Some of the best candidate promoters rely on a synthetic molecule for repression, such as the allolactose analog, isopropyl ß-D-1-thiogalactopyranoside (IPTG), and the tetracycline analog anhydrotetracycline (aTc). While these have been effective in controlling gene expression and are unlikely to be encountered in significant concentrations in nature, the addition of these to large-scale cultures may be a significant expense.

It is important to choose robust signals for induction of the selected containment system. The signal response (cell death) should be strong when the cell exists outside the lab or industrial setting. In the production setting, expression of lethal genes must be minimized to reduce the loss in productivity that is expected to result. Metal ion-inducible promoters such as *PnrsB* (Ni_2_^+^-inducible) have been used frequently for this purpose because they are tightly repressed in the absence of metal ions (Englund et al., 2016). This tight repression is an important property when expressing toxic genes (Cheah et al., 2013). However, with this promoter and others like it, toxic gene expression would be repressed in most natural waters because they have low concentrations of metal ions. It may be possible to invert this signal by using the metal ion-inducible promoter to drive expression of a repressor transcription factor that acts on the promoter of the toxic gene. It is unclear how this might impact the leakiness of the lethal gene expression.

#### Types of Lethal Genes

A variety of lethal genes have been utilized in cyanobacteria—both for biocontainment and for counterselection including proteins from toxin-antitoxin systems and phage lysis proteins (Cheah et al., 2013; Čelešnik et al., 2016; Zhou et al., 2019). An important benefit to using toxins from antitoxin systems is that the antitoxin can be co-expressed at a low level (or induced only in the lab/industrial setting) to prevent leaky expression of the toxic protein from reducing growth rates in the lab.

Thousands of toxin-antitoxin systems have been identified, and this remains an active area of research in microbiology (Page and Peti, 2016). Such systems may have diverse roles in their native context. They were hypothesized to be important to plasmid maintenance by the mechanism known as post-segregation killing (Gerdes et al., 1986). In such systems, the antitoxin is encoded on a plasmid and the toxin in the chromosomal DNA. Cells that do not inherit a copy of the plasmid with the antitoxin are killed by the expression of the toxin alone. More recently, they have been suggested to be important in inducing a persistence state in poor environmental conditions (Gerdes, 2016; Page and Peti, 2016). Under normal growth conditions, the expression of the antitoxin exceeds the expression level of the toxin. A stressor, such as nutrient starvation or antibiotics exposure, perturbs this balance and the toxin expression exceeds that of the antitoxin, leading to growth arrest which can be reversed when more favorable conditions return. Some work has suggested that persistence is stochastically induced within populations (Verstraeten et al., 2015), which can be beneficial in surviving infrequent, severe, and difficult-to-sense stresses (Kussell and Leibler, 2005).

Page and Peti (2016) provide an excellent review of the evidence that at least some toxin-antitoxin systems are used in this way (Page and Peti, 2016). They classified systems according to the mechanism of action of the toxin gene and the mechanism by which the antitoxin can inactivate the toxin. Toxins may halt metabolism by depolarizing membranes, prevent production of new proteins by wholesale degradation of mRNA, or degrading the already existing proteins. The potential reversibility of any of these types of toxins may make them less attractive for biocontainment strategies. Generally, it is not known how long a cell can persist while these toxins are active and this performance parameter has not typically been measured in biocontainment reports.

Nucleases that degrade the chromosomal DNA might be more advantageous because the mechanism of killing the cell also degrades the recombinant DNA and may prevent horizontal gene transfer to or from the GMO. Restriction enzymes, Cas9, and nucleases that are used to scavenge nucleotides from the environment are all candidates for this category. Interestingly, EcoRI has been used in a biocontainment module in *Escherichia coli* despite the fact that *E. coli* DNA should be protected from EcoRI by the native methylation pattern. Apparently, overexpression of the restriction enzyme can easily overcome the protection offered by the native methylation and cause cell death by DNA degradation (Chan et al., 2016).

Some nucleases are used by cells to scavenge nucleotides from the environment. The NucA nuclease from *Serratia marcescens* is one such protein which has been used for biocontainment (Balan and Schenberg, 2005). This nuclease is activated by disulfide bond formation which does not occur in the reducing environment inside the cell. Only when it is secreted into the oxidizing environment outside the cell does it become active. The organism’s own nucleotides are thus protected (Benedik and Strych, 1998). This mechanism of inactivation may limit the utility of this nuclease in biocontainment strategies. Another nuclease, NucA from *Anabaena* sp. PCC 7120, is inactivated by dimerization with a specific inhibitor protein, NuiA (Meiss et al., 2000). In this case, by careful selection of promoters, NucA and NuiA can be co-expressed as part of a biocontainment module such that NucA is inactivated by NuiA in the lab, but NucA expression exceeds that of NuiA when the cell escapes into the environment. Since NucA is secreted by *Anabaena*, it may also be important to identify and remove any signal peptides that target the protein to the extracellular space so it can effectively degrade the DNA and RNA inside the cell.

#### Genetic Instability

Mutations within toxic protein coding sequences that may result in inactive protein or reduced toxicity are a serious threat to the efficacy of biocontainment modules. Horizontal gene transfer from other organisms potentially can complement knockouts made for synthetic auxotrophy strategies. The natural competence of some cyanobacteria to uptake DNA increases the probability that this may occur. In some species, the pili gene *hfq* is essential to natural competence (Dienst et al., 2008), and in one study, the natural competence genes were knocked out in order to maintain the synthetic auxotrophy (Clark et al., 2018). This suggests one method to avoid possible failure of the genetic biocontainment module. Mutation hot spots within toxin gene should be avoided if possible (Rogozin and Pavlov, 2003). Overlapping the coding sequence of the toxin gene with an essential gene has been proposed as a method for selecting against mutations, though the process of designing and testing such intertwined coding sequence presents a formidable challenge (Blazejewski et al., 2019). Toxin genes used in the reports discussed below have generally been chromosomally integrated rather than maintained on replicating plasmids which may easily be lost, especially if there is selective pressure against them. Genes needed to facilitate growth through synthetic auxotrophy may be maintained on replicative plasmids. However, this may facilitate the spread of those genes to possible contaminating species through plasmid transfer.

### Specific Examples of Genetically Encoded Biocontainment Modules in Cyanobacteria

In this section, we discuss the recent successes in developing biocontainment modules in cyanobacteria. Table 1 summarizes these studies.

**Table 1:** A summary of recent reports which have tested biocontainment strategies in cyanobacteria.

Strain	Type	Promoter	Induction	Genes/proteins	Escape frequency	Reference
*Synechocystis* sp. PCC6803	Toxin-antitoxin	*PcopM*	Zn_2_^+^	NucA/NuiA (from *Anabaena*)	“Complete autodestruction upon Zn_2_^+^ induction”	Čelešnik et al., 2016
*Synechocystis* sp. PCC6803	Toxin-antitoxin	*PnrsB*	Ni_2_^+^ or Co_2_^+^	NucA/NuiA (from *Anabaena*)	Weak autotoxicity	Čelešnik et al., 2016
*Synechocystis* sp. PCC6803	Toxin-antitoxin	*PcopB*	Zn_2_^+^	ssr1114/slr0664	Weak autotoxicity	Čelešnik et al., 2016
*Synechocystis* sp. PCC6803	Toxin-antitoxin	*PcopB*	Zn_2_^+^	slr6101/slr6100	Weak autotoxicity	Čelešnik et al., 2016
*Synechocystis* sp. PCC6803	Toxin-antitoxin	*PrnpB*	Constitutive (antitoxin induced by Zn_2_^+^)	ssr1114/slr0664	Weak autotoxicity on metal ion withdrawal (antitoxin expressed using P*copB*)	Čelešnik et al., 2016
*Synechococcus* sp. PCC7002	Synthetic auxotrophy			High CO_2_ dependence (CCM deletion)	<1 × 10^−9^/CFU	Clark et al., 2018
*Synechococcus elongatus* PCC7942	Synthetic auxotrophy			Phosphite dependence	Below detection limit over 28 days (3.6 × 10^−11^ per CFU)	Motomura et al., 2018
*Synechococcus* sp. PCC7002	Growth on melamine	*Pc223*	Constitutive	Synthetic melamine utilization operon	Could be converted to synthetic auxotrophy strategy if ammonia and nitrate uptake inhibited	Selão et al., 2019
*Synechococcus* sp. PCC7002	Growth on phosphite	*PpsbA (A. hybridus)*	Constitutive	PtxD from *Pseudomonas stutzeri* WM88	Could be converted to synthetic auxotrophy strategy if phosphate uptake inhibited	Selão et al., 2019
*Synechococcus elongatus* PCC7942	Toxin-antitoxin	*PisiAB*	Reduced iron availability	SepA2/SepT2	<1 × 10^−9^/CFU	Zhou et al., 2019
*Synechococcus elongatus* PCC7942	Toxin-antitoxin	*PisiAB*	Reduced iron availability	SepA1/SepT1	Weak autotoxicity	Zhou et al., 2019
*Synechococcus elongatus* PCC7942	Toxin-antitoxin	*PisiAB*	Reduced iron availability	slr6101/slr6100	Weak autotoxicity	Zhou et al., 2019
*Synechococcus elongatus* PCC7942	Toxin-antitoxin	*PisiAB*	Reduced iron availability	ssr1114/slr0664	Limited growth	Zhou et al., 2019
*Synechococcus elongatus* PCC7942	Membrane disruption	*PisiAB*	Reduced iron availability	P22 phage holin-endolysin	Slightly reduced growth in induction media	Zhou et al., 2019
*Synechococcus elongatus* PCC7942	Toxin-antitoxin	*PisiAB*	Reduced iron availability	NucA/NuiA (from *Anabaena*)	Growth arrest after 24 h	Zhou et al., 2019
*Synechococcus elongatus* UTEX2973	Toxin-antitoxin	*PisiAB*	Reduced iron availability	SepA2/SepT2	<1 × 10^−9^/CFU	Zhou et al., 2019
*Synechococcus elongatus* UTEX2973	Toxin-antitoxin	*PisiAB*	Reduced iron availability	SepA1/SepT1	Weak autotoxicity	Zhou et al., 2019

#### Toxic Proteins

Čelešnik et al. (2016) tested several toxin proteins in *Synechocystis* sp. PCC6803 (S. 6803). They focused on metal ion-inducible promoters including the zinc-inducible *PcopB* and *PcopM*, and nickel-inducible *PnrsB* promoters, which tend to be tightly controlled and not leaky. *PnrsB* had previously been used by others to control the expression of the toxin *mazF* gene which could be used as a counterselection marker (Cheah et al., 2013). Toxin proteins included the NucA nuclease from *Anabaena*, which is used to scavenge nucleic acids from the environment and can degrade single and double-stranded DNA and RNA (Meiss et al., 1998). Along with NucA, two other native toxin-antitoxin systems from S. 6803 were tested with the same three metal ion-inducible promoters. The P*copM*-NucA combination resulted in growth arrest in liquid culture, loss of viability in a tetrazolium assay (a measure of reducing capacity), and no growth on agar plates when induced by zinc, showing the toxicity of this gene. Strains with *PnrsB*-driven NucA and *PcopB*-driven slr0664 (a putative RNase and relative of RelE from *E. coli*; Ning et al., 2011) were still capable of growing, though at a slower rate. In each strain, the antitoxin was co-expressed to avoid growth defects that may arise due to leaky expression. As the authors point out, metal ion concentrations in natural waters tend to be much lower than the ~4–20 μM concentrations needed to induce these promoters. They, therefore, tested another design which put the antitoxin of the slr0664 toxin under control of the *PcopB* promoter and the toxin under control of the constitutive *PrnpB* promoter. This strain showed reduced growth compared to the wild type when grown in standard BG11 and similar growth to wild type when grown in BG11 supplemented with 4 μM zinc (Čelešnik et al., 2016). It has been shown by others that slr0664 is activated by proteolysis of the antitoxin, ssr1114, which may be dependent on growth conditions (Ning et al., 2011), and it is unclear whether this layer of regulation affected the outcome of this experiment.

Zhou et al. (2019) took a similar approach in designing biocontainment modules for *S. elongatus* UTEX 2973 and PCC 7942. They also tested NucA from *Anabaena*, the holin and endolysin from P22 phage, and a native RNase, SepT2, that is part of a toxin-antitoxin system. The holin and endolysin had been previously used to facilitate nickel-inducible lysis of *Synechocystis* sp. PCC 6803 using the *PnrsB* promoter (Liu and Curtiss, 2009). Zhou et al. (2019) first screened several metal ion-inducible promoters and selected a low-iron-inducible promoter, *PisiAB* from S. 7942, which was expected to be induced by the low-iron content of most water in the natural environment. For SepT2 and NucA, the respective antitoxins were co-expressed under control of the native P*psbA2* promoter. The phage lysis proteins reduced growth slightly even when uninduced (iron-replete condition) and minimally reduced growth further when induced by low-iron concentrations. The NucA gene caused growth arrest within 24 h of a shift to low-iron media. The toxin-antitoxin systems ssr1114/slr0664, slr6101/slr6100, and SepA1/SepT1 were found to be bacteriostatic rather than lethal. SepT2 was found to be effective in both S. 2973 and S. 7942 with colony forming units below the detection limit of 10^−9^ after 3 days of low-iron media exposure (Zhou et al., 2019).

#### Synthetic Auxotrophy

Algae have been engineered to be dependent on chemical species that are in lower abundance in the environment than what is needed to sustain growth (Figure 1B). Synthetic auxotrophs that are dependent on high carbon dioxide concentrations, phosphite, and melamine or urea for growth have been examined. Unlike the lethal gene strategies, synthetic auxotrophy may be less likely to be overcome by mutations; while a single point mutation may inactivate a lethal gene, horizontal gene transfer from other organisms may be necessary for synthetic auxotrophs to regain the ability to utilize more common nutrients.

Phosphate is the form of phosphorous typically used to grow cyanobacteria. Some organisms can utilize phosphite, and the genes responsible for transport and oxidation of phosphite to phosphate are known. These genes can be introduced to cyanobacteria, and they can be grown in media lacking phosphate, which has been shown to be effective in reducing biological contamination (González-Morales et al., 2020) since not all organisms can utilize phosphite. Selão et al. (2019) completed similar work in creating strains of cyanobacteria that were auxotrophic of phosphite, and melamine or urea for nitrogen sources. To make *Synechococcus* sp. PCC7942 a synthetic auxotroph of phosphite, the transporter genes *HtxBCDE* and the phosphite oxidation gene, *ptxD*, from *Pseudomonas stutzeri* WM88 were introduced, and the phosphate transporters knocked out. The escape frequency was found to be undetectable over 28 days with a detection limit of 3.6 × 10^−11^ per colony forming unit. Although natural water may contain low levels of phosphite, the engineered strain was not able to grow in BG11 media prepared with sterilized fresh water from a natural source (with or without phosphate). The potential for horizontal gene transfer was assessed by growing the engineered strain with the wild-type *Synechocystis* sp. PCC6803. The co-culture was plated on selective media for the kanamycin resistance cassette, which was used to disrupt the phosphate transporter. All 28 colonies screened were found to be S. 7942 rather than the WT S. 6803 (Motomura et al., 2018). Similar efforts to generate synthetic auxotrophies of nitrogen sources could build on the work of Selão et al. (2019) by knocking out ammonium transporters.

Although there are not many examples yet of biocontainment developed for eukaryotic algae, we expect that many of the strategies effective in cyanobacteria may also be effective when used for biocontainment of eukaryotic algae. Growth on phosphite has also been demonstrated in the fast-growing eukaryotic algae, *Picochlorum* (Dahlin and Guarnieri, 2022). In *Chlamydomonas reinhardtii*, the chloroplast genome does not use the UGA codon (the opal stop codon); leading Young and Purton (2016) to suggest that it may be utilized for biocontainment. Specifically, they suggested that UGA can be inserted into an essential gene, and the chloroplast transformed to express the corresponding tRNA synthetase that utilizes a nonnatural amino acid. Such a strain would be dependent on external supply of the nonnatural amino acid for growth (Young and Purton, 2016). In a follow-up study, this group expressed the *ptxD* gene for phosphite utilization, incorporating the UGA codon substitution, and co-expressing a tRNA with its anticodon modified to decode this codon. This design was not intended to render the modified strain dependent on a nonnatural amino acid, but the authors considered this a strategy for reducing the possibility of the ptxD gene being transferred to other organisms, and, thus, another form of biocontainment (Changko et al., 2020).

A pair of publications report on creating strains of cyanobacteria which are dependent on high carbon dioxide concentrations for growth by knocking out the genes needed for the CCM. Lee et al. (2021) knocked out the carbon concentrating mechanism *in S. elongatus* PCC7942. They observed no growth of this strain when grown in photobioreactors with less than 5% CO_2_ in the sparge gas. However, even in elevated CO_2_, the strain grew more slowly than wild type and a loss in productivity of the target molecule, farnesene, was also observed. Recovery from these losses was achieved by complementing the strain with a bicarbonate transporter and carbonic anhydrase genes. This strain could still grow, though at a diminished rate, when sparged with air (Lee et al., 2021).

Clark et al. (2018) also knocked out the carbon concentrating mechanism genes of *Synechococcus* sp. PCC7002 to create a strain dependent on high CO_2_ for growth. Measuring colony forming units to demonstrate compliance with the NIH escape frequency guideline, they showed that the guideline could be met in ambient air growth. Using co-cultures of the CCM knockout with the wild type, it was shown that the guideline threshold may be exceeded *via* horizontal gene transfer. Knockout of a gene essential for horizontal gene transfer was effective in reducing the escape frequency down below the guideline threshold. Henley et al. (2013) suggested the potential for horizontal gene transfer from the GE algae to wild organisms, which are carried into the pond—this paper suggests one effective approach to address this risk. While this knockout reduced the frequency of acquisition of the CCM genes in co-culture, it did not appear to change the frequency of gene transfer from the engineered cells to their co-culture partners (Clark et al., 2018).

### Future Directions

Based on studies summarized above, researchers have been quite successful in demonstrating that both toxic genes and synthetic auxotrophy can be effective in reducing escape frequencies to below the NIH guideline. In some cases, growth rates were only reduced, and escape frequencies exceeded the NIH guideline. Researchers have not always examined why some strategies failed. Inspection of the failure mechanism(s) would benefit development of future strategies. There may be many possible explanations. Was the expression level of the toxin protein insufficient to overcome the expression level of the co-expressed antitoxin? Did some cells in the culture mutate to reduce the activity of the toxin? Mechanistic understanding of biocontainment efficacy, and the impact upon strain fitness, will ultimately enable predictive design to concurrently maximize biocontainment and bioproductivity.

Given that most tests have relied on counting colony forming units, it may be beneficial to examine whether growth on agar plates in the lab is more, or less, permissive to growth than conditions outside the lab. Fewer stressors may be present in the controlled environment of the lab, but it may be possible for escaped cells to find some ecological niche in the natural environment where the molecule needed to repress a toxin gene can be found. Testing of biocontainment strategies in more realistic environmental conditions would be beneficial to our understanding of the efficacy of genetically encoded biocontainment systems. For example, engineered strains could be grown in media that models the natural environment that the cells are likely to encounter if they physically escape. Growth media could also be developed to demonstrate the escape frequency in a worst-case scenario. For a synthetic auxotrophy strategy, this media would include possible alternative nutrients, or the maximum known environmental concentration of the nutrient for which the algae has been made dependent. A further limitation of present tests of these systems is that they tend to only measure growth or no growth in one condition. It may be possible for cells expressing toxin proteins to persist for some period of time, with the potential for revival. If there is any chance that the kill switch signal can be reversed in the natural environment, the duration that the cells can persist with the switch “on” should be determined.

In some cases, it may be possible for escaped cells to experience fluctuations in the environmental signal needed to kill the cell. Another question that may be important to probe is how long must the genetic kill switch be “on” for it to result in complete killing of all cells? Can they recover if the switch is not “on” for long enough? Is it possible for such biocontainment strategies to give rise to persistence in the environment? It is generally assumed that the lab conditions used to test biocontainment strategies are stringent in that the cells are grown without competitors or predators and in media that is richer in nutrients than most natural waters, with light intensity that is not too high or too low and does not include UV radiation. However, the complexity of the natural environment may provide some opportunities for escape that is not represented in such tests. For example, contact with a multitude of other organisms could give the cells the opportunity to obtain the signal molecule which represses the toxic gene or which the synthetic auxotrophy has made the cell dependent on *via* cross-feeding. Some of the signal molecules may be present in sufficient concentrations in some environments to prevent efficient killing of the cells. Impacts of biocontainment strategies to productivity should also be assessed because they are unlikely to be implemented if they reduce productivity. Further genetic modifications, beyond synthetic auxotrophy and toxic gene strategies, which improve fitness in the cultivated setting but decrease fitness in the natural environment should also be identified to further reduce escape frequencies. The Standards in Synthetic Biology website^3^ is currently collecting biocontainment strategies that have been tested and may be applied to GE algae.

## Author Contributions

JS drafted the manuscript. WX, MG, and JY edited and provided feedback on the manuscript. All authors contributed to the article and approved the submitted version.

## Funding

This material is based upon work supported by the U.S. Department of Energy, Office of Science, Office of Biological and Environmental Research, Genomic Science Program under Secure Biosystems Design Science Focus Area (SFA), IMAGINE BioSecurity: Integrative Modeling and Genome-scale Engineering for Biosystems Security, under contract number DE-AC36-08GO28308. The views and opinions of the authors expressed herein do not necessarily state or reflect those of the United States Government or any agency thereof. Neither the United States Government nor any agency thereof, nor any of their employees, makes any warranty, expressed or implied, or assumes any legal liability or responsibility for the accuracy, completeness, or usefulness of any information, apparatus, product, or process disclosed, or represents that its use would not infringe privately owned rights.

## Conflict of Interest

The authors declare that the research was conducted in the absence of any commercial or financial relationships that could be construed as a potential conflict of interest.

## Publisher’s Note

All claims expressed in this article are solely those of the authors and do not necessarily represent those of their affiliated organizations, or those of the publisher, the editors and the reviewers. Any product that may be evaluated in this article, or claim that may be made by its manufacturer, is not guaranteed or endorsed by the publisher.

## Footnotes

^1^ https://www.energy.gov/sites/prod/files/2019/08/f65/ea-1786-fea-2010.pdf

^2^ https://www.epa.gov/sites/default/files/2020-10/documents/algae_supplement_091420.pdf

^3^ https://standardsinsynbio.eu
